# Molecular Epidemiology of Dengue in Panama: 25 Years of Circulation

**DOI:** 10.3390/v11080764

**Published:** 2019-08-20

**Authors:** Yamilka Díaz, María Chen-Germán, Evelia Quiroz, Jean-Paul Carrera, Julio Cisneros, Brechla Moreno, Lizbeth Cerezo, Alex O. Martinez-Torres, Lourdes Moreno, Itza Barahona de Mosca, Blas Armién, Rubing Chen, Nikos Vasilakis, Sandra López-Vergès

**Affiliations:** 1 Department of Research in Virology and Biotechnology, Gorgas Memorial Institute of Health Studies, Justo Arosemena avenue and 35st street, 0816-02593 Panama, Republic of Panama; 2 Faculty of Medicine, University of Panama, 3366 Panama 4, Republic of Panama; 3 National Department of Epidemiology, Ministry of Health, 0816-06812 Panama 1, Republic of Panama; 4 Department of Microbiology and Parasitology, University of Panama, 3366 Panama 4, Republic of Panama; 5 Department of Research in Emergent and Zoonotic diseases, Gorgas Memorial Institute of Health Studies, Justo Arosemena avenue and 35st street, 0816-02593 Panama, Republic of Panama; 6 Research Direction, Universidad Interamericana de Panama, Apto., 0830-00929 Panama, Republic of Panama; 7 Department of Pathology and Center for Biodefense and Emerging Infectious Diseases, University of Texas Medical Branch, 301 University Blvd, Galveston, TX 77555-0609, USA; 8 Center for Tropical Diseases, University of Texas Medical Branch, 301 University Blvd, Galveston, TX 77555, USA; 9 Institute for Human Infections and Immunity, University of Texas Medical Branch, 301 University Blvd, Galveston, TX 77555-0610, USA; 10 The World Reference Collection of Emerging Viruses and Arboviruses, University of Texas Medical Branch, 301 University Blvd, Galveston, TX 77555-0609, USA

**Keywords:** Dengue virus, molecular epidemiology, Flavivirus, the Americas, Panama, outbreak, arbovirus

## Abstract

Dengue virus (DENV) is the most prevalent arbovirus in terms of human public health importance globally. In addition to DENV epidemiological surveillance, genomic surveillance may help investigators understand the epidemiological dynamics, geographic distribution, and temporal patterns of DENV circulation. Herein, we aimed to reconstruct the molecular epidemiology and phylogeny of DENV in Panama to connect the epidemiological history of DENV dispersal and circulation in Latin America. We retrospectively analyzed the epidemiological data obtained during 25 years of DENV surveillance in Panama. DENV was reintroduced in Panama in 1993 after a 35 year absence of autochthonous transmission. The increase in the number of total dengue cases has been accompanied by an increase in severe and fatal cases, with the highest case fatality rate recorded in 2011. All four serotypes were detected in Panama, which is characterized by serotype replacement and/or co-circulation of multiple serotypes. Phylogenetic analysis of datasets collected from envelope (E) gene sequences obtained from viruses isolated from human sera demonstrated that circulating viruses were highly diverse and clustered in distinct clades, with co-circulation of clades from the same genotype. Our analyses also suggest that Panamanian strains were related to viruses from different regions of the Americas, suggesting a continuous exchange of viruses within the Americas.

## 1. Introduction

Dengue viruses (DENV) are arthropod-borne viruses of the genus *flavivirus*, family *Flaviviridae*, with four currently recognized serotypes (DENV-1–4) [1,2]. The principal mosquito vectors of transmission are members of the *Aedes* genus [3]. Dengue disease, historically described as "break-bone fever", can induce fever, headache, myalgia, fatigue, and in rare cases, severe dengue disease (SDD), presenting with hemorrhage, plasma leakage, and death [4]. DENV has been present in the Americas at least since the 17th century [5]. Panama formally reported dengue-like illness in 1904, with subsequent outbreaks [3,6]. Aggressive and sustainable vector control programs led to the eradication of *Aedes aegypti* in the Americas from 1947–1970 [3], however following the reintroduction of *Aedes aegypti* and then the dengue virus in the late 1970s and early 1980s, the total dengue cases in the Americas increased exponentially [7,8,9]. Panama remained *Aedes aegypti*-free until their re-infestation in 1985 [3], and it was the last country in Central America to detect autochthonous dengue cases, reporting cases of DENV-2 in 1993 [10]. Since then, all four serotypes have been continuously circulating and causing outbreaks. 

The clinical manifestations of dengue and their severity depend on various factors, such as the genetics of the virus and the host, as well as previous exposure to heterotypic DENV infections [11]. Each DENV serotype consists of various genotypes [12], representing their great diversity and allowing for the use of molecular epidemiology as a tool for understanding their evolution and transmission dynamics [13,14,15]. In recent years, the increase of dengue cases in the Americas has been associated, in part, with the co-circulation of several serotypes and the emergence of new lineages, replacement, or co-circulation of different clades in the same genotype [15,16,17]. However, in Central America and the Caribbean, few countries have analyzed the genetics of the sampled DENV strains and their contribution to transmission dynamics [18,19]. 

There is limited information on the spatiotemporal circulation of various serotypes and genotypes in Panama [19,20]. We previously demonstrated that the DENV-2 strains detected in the 2011 Panama outbreak that were associated with a high case fatality rate [21] were related to strains from outbreaks in Nicaragua and Guatemala in 2009–2010 [22]. In order to determine the effects of co-circulation and the introduction of new DENV serotypes in Panama, we analyzed the dynamics of the strains sampled over a period of 25 years, complemented with an analysis of epidemiological data. Our study provides insight into DENV circulation in Panama, enhancing our understanding of the geographical distribution of DENV serotypes, genotypes and clades in the region. 

## 2. Materials and Methods

### 2.1. Bioethical Approval

The Institutional Research Bioethics Committee approved this study under approval code 019/CBI/ICGES/18 on January 11th 2018, to genetically analyze DENV strains from the virus repository of Gorgas Memorial Institute for Health Studies (ICGES) isolated from sera of febrile patients obtained through the Dengue surveillance program from ICGES and the Ministry of Health of Panama (MINSA) from 1993 to 2017. This IRB permit allowed us to analyze de-identified epidemiological data from these years collected by the National Department of Epidemiology (NDE) from MINSA.

### 2.2. Case Definition

The MINSA used the 1997 World Health Organization (WHO) clinical classification of dengue fever (DF), dengue hemorrhagic fever (DHF), and dengue shock syndrome (DSS) from 1993 to 2011, whereas dengue with or without warning signs and severe dengue WHO 2009 classification has been used since 2012. Executive order N 1617 issued in 2014 regulates the mandatory notification of the dengue suspected cases to the NDE from public and private institutions and the mandatory use of WHO’s dengue suspected case definition [23].

### 2.3. National Dengue Laboratory Surveillance Program

The National dengue surveillance program was established in 1988 by MINSA, in collaboration with ICGES, to survey the entomological situation of *Aedes* spp. vectors as well as all dengue human clinical cases in the country [10]. Clinical suspected cases were confirmed by laboratory diagnosis or epidemiological link. The epidemiological link was defined as a clinical dengue suspected case in close contact (home or workplace) with a laboratory dengue confirmed case. This contact had to happen within less than 30 days of the onset of symptoms of the confirmed case. From 1993 to 2009, ICGES received all serum samples from dengue suspected cases for laboratory confirmation. Acute sera samples (0–3 days post onset of symptoms) were analyzed using viral isolation by the inoculation of Vero cells (African green monkey-ATCC CCL-81, USA). When cytopathic effect (CPE) was evident or at 14 days post-inoculation, the supernatant was collected and stored at −80 °C in the ICGES viral repository. To characterize the serotype of the isolated virus, indirect immunofluorescence assay (IFA) using monoclonal antibodies supplied by the Centers for Disease Control and Prevention (CDC) Fort Collins was performed. Convalescent serum samples (5–20 days post-symptom onset) were analyzed using capture enzyme-linked immunosorbent assay (ELISA) for Dengue IgM/IgG (Panbio, Abott laboratories). In 2010, the national surveillance program decentralized dengue surveillance, establishing capacity in all main hospitals and clinical units around the country for serological tests (ELISA IgM/IgG) used for laboratory confirmation of convalescent samples and NS1 ELISA for confirmation of acute samples. ICGES’ leadership in supporting national surveillance efforts continues to this day with the screening of acute samples, virus isolation, and serotype typing through the CDC qRT-PCR system [24]. 

### 2.4. Epidemiological Data and Statistical Analysis

Epidemiological details of dengue cases were acquired using the databases from the National Dengue Surveillance Program from 1999 to 2017. To account for comparability between previous (1993–2012, DF, DHF, DSS) and newer (2012–2017, dengue without and with warning signs, severe dengue) dengue case definitions, we used the criteria applied by MINSA to normalize the categories. Consequently, the outcome variables were reduced into two categories: (i) classic dengue, which covers dengue without warning signs; and (ii) hemorrhagic dengue, which encompasses dengue with warning signs, severe dengue, DHF, and DSS. Independent variables included were age group (0–10, 11–20, 21–30, 31–40, 41–50, 51–60, 61–70, 71–80, >80), gender, and geographic regions (Panama provinces and indigenous regions). Due to the low number of dengue cases in indigenous regions, we arbitrarily included the indigenous regions within their closest provinces (Guna Yala indigenous region with Darien province, Ngöbe-Buglé indigenous region with Chiriqui province) (Supplementary Figure S1). We compared those with classic dengue to those with hemorrhagic dengue using the following variables: gender, age group, and geographic region. Descriptive analysis was obtained for each variable. The association at the univariate and multivariate level between the outcome and independent variables was expressed as odds rations (ORs) using an unconditional logistic regression model. For statistical analysis, reference groups were selected using the categories with a higher number of observations, and *p* values < 0.05 were considered significant. ORs > 1 indicated a higher risk compared to the reference group, ORs < 1 indicated a protective factor (lower risk) compared to the reference group. All statistical analyses were conducted using STATA 14.1 (STATA Corporation, College Station, Texas, US).

### 2.5. Sample Selection and E Gene Sequencing

We selected, when possible, at least one viral isolate per year, per serotype, per province, and per month from the ICGES repository, resulting in the analysis of a total of 222 strains. 

Viral RNA from each isolate was extracted using QIAmp viral RNA mini kit (QIAGEN, Hilden, Germany). RT-PCR with serotype-specific primers covering the envelope (E) gene (primers designed by [25,26], and UTMB, Supplementary Table S1) was performed using the Titan kit (Sigma-Aldrich, St. Louis, MO, USA), or Platinum Taq DNA polymerase high fidelity (Thermo Fisher Scientific, Waltham, MA, USA) generating amplicons of approximately 1800 bp. Amplicons were then purified using QIAquick PCR purification kit (QIAGEN), and sequences were obtained through Sanger sequencing using BigDye terminator v3.1 (Applied Biosystems, Foster city, CA, USA) in an Applied Biosystems 410 Genetic Analyzer following the manufacturer’s protocols. Overlapping sequence segments were assembled and analyzed using Sequencher (version 5.0.1). Of the selected 222 strains, the full E gene was successfully obtained for 179 isolates and deposited in GenBank (93 strains for DENV-1: MH879156–MH879248; 53 for DENV-2: KY977454–KY977459, MF491509, MF491510 [21], and MH824748–MH824794; 26 for DENV-3: MH925274–MH925300; and 7 for DENV-4: MH824741–MH824747). 

### 2.6. Sequencing and Phylogenetic Analyses

Available sequences for each DENV serotype were downloaded from GenBank [27] and from the NIAID Virus Pathogen Database and Analysis Resource [28] in order to construct datasets for phylogenetic analysis. All E gene sequences from these databases and from Panama (DENV-1: 2188 total sequences; 2179 for DENV2; 642 for DENV-3; 921 for DENV-4) were aligned using SEAVIEW 4.0 software [29]. Model selection for each dataset was carried out using the Model Compare package from MEGA7 [30]. Identical sequences from the same year and date and same country and location were deleted using the Neighbor-Joining (NJ) tree topology without sacrificing the overall genetic diversity. We also constructed smaller datasets using the NJ tree, with representative sequences from each genotype and/or clade, but we kept all sequences close to the Panamanian sequences and all Panamanian strains: 182 sequences for DENV-1, 153 for DENV-2, 97 for DENV-3, and 48 for DENV-4 (Supplementary Table S2). We then performed a Maximum Likelihood (ML) estimation to reconstruct the phylogenetic history of all DENV serotypes using the General Time Reversible with proportion of invariable sites and gamma distribution (GTR + I + gamma). Phylogenetic trees were visualized using the midpoint root for clarification. The statistical significance of the tree topology was evaluated by bootstrap resampling of the sequences 1000 times with the RaxML algorithm using MEGA7. The protein E amino acid sequences were obtained from the translation of E gene and analyzed using MEGA7.

## 3. Results

### 3.1. Epidemiological Analysis

Since the DENV re-emergence in Panama in 1993 to 2017, a total of 67,834 dengue cases were reported to MINSA, with 2.9% (1974 cases) classified as hemorrhagic dengue and 78 resulting in death (Table 1). During this period, several outbreaks occurred, most notably the outbreaks of 2005–2006 (incidence rates of 170/100,000 inhabitants in 2005 and 131.7 in 2006), 2009, with the highest incidence rate (216.5), and 2013–2014 (124.2 and 141, respectively). Between 1999 and 2017, the Dengue incidence rate was higher in patients between 30 to 59 years old; however, during recent years, the age group under 19 years old has had the highest incidence, including years 2010, 2012, 2016, and 2017 (Supplementary Table S3). 

From 1999 to 2017, 68.0% (39254/57691) of dengue cases were confirmed by at least one of the following laboratory methods: viral isolation, immunofluorescence, RT-PCR, IgM, or NS1 ELISA (Table 1). The rest of the cases were confirmed by epidemiological link. 

**Figure 1:**
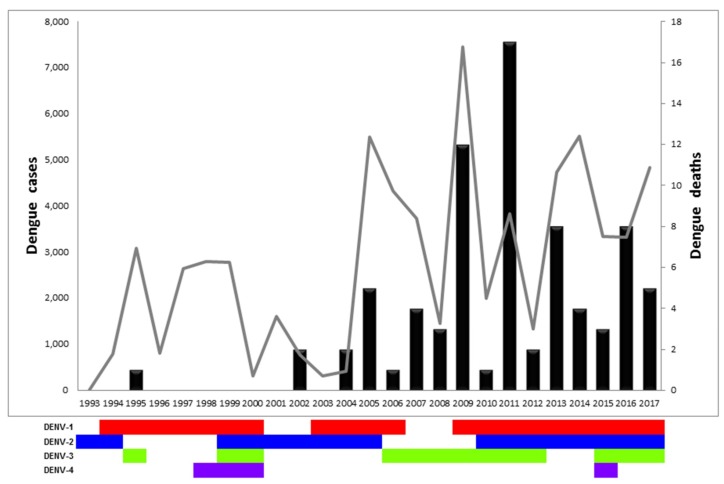
Dengue cases, dengue deaths, and circulating serotypes in Panama from 1993 to 2017. Graph representing the number of dengue cases (gray line, left Y axis), dengue deaths (black bars, right Y axis), and circulating serotypes per year (DENV-1 in red, DENV-2 in blue, DENV-3 in green, DENV-4 in violet).

Although hospitalization before 2005 was not recorded, our database from 2005 to 2017 indicates that 8.26% (4254/51494) of cases required hospitalization (Table 1). From 2005 to 2011, the numbers of hospitalized cases increased during 2006, 2009, and 2011. After implementation of the 2009 WHO clinical dengue classification, the number of hospitalized cases steadily increased. There were no statistical differences between age groups that required hospitalization, even though the groups of less than 1 year of age and greater than 50 years of age represented a smaller percentage of all hospitalized cases (1.5% for <1 year old and 8.6%, 7.6%, and 10% for 50–59, 60–69, and >70 years old age groups, respectively) (Supplementary Table S4). The age groups 10–19 and 20–29 years old had a slightly higher percentage of hospitalized cases (19.1% and 15.8%, respectively). Before the 2009 outbreak, Dengue deaths were sporadic, even when fatal cases were reported every year after 2004, with a total of 18 cases from 1993 to 2008. However, from 2009 to 2017, 60 fatal cases were due to dengue infection, amounting to 76.9% of all recorded dengue associated deaths from 1993–2017 (Table 1). The case fatality rate (CFR) differed between outbreaks: the highest was in 2004 (0.49/100,000 inhabitants), followed by the 2011 outbreak (0.44/100,000 inhabitants.). Further, 2011 was also the year with the most fatal cases (Table 1, Figure 1; [21]). 

The risk of developing dengue hemorrhagic fever was independent of gender (Table 2). At the univariate level analysis for age groups, compared with the reference group (11–20 years old), the risk of hemorrhagic dengue increased in the 0–10 years-old age group 1.36 times (95% CI: 1.17–1.58, *p* < 0.001). While being in the 31–40 and 41–50 age groups decreased the risk of developing dengue hemorrhagic fever, with 0.81 (95% CI: 0.70–0.95, *p* = 0.009) and 0.77 (95% CI: 0.65–0.90, *p* = 0.002), respectively. After adjustment by gender and region at the multivariate level, age groups 31–40 and 41–50 still had a protective factor for developing dengue hemorrhagic fever compared to the reference group. At the multivariate level, the risk of developing hemorrhagic fever was still increased 1.33 times (95% CI: 1.14–1.55, *p* < 0.001) at 0–10 years old. When analyzed at the univariate level, the risk of developing hemorrhagic fever within the 71–80 year-old age group increased 1.29 times, with a *p* value of 0.056. At the multivariate level, the risk of developing hemorrhagic fever was 1.33 higher than the reference group, with a *p* value of 0.033. 

The geographic risk of developing dengue hemorrhagic fever compared with the reference region (Metropolitan) at the univariate level, have a protective factor in Chiriquí 0.29 (95% CI: 0.22–0.38, *p* = < 0.001), Coclé 0.32 (95% CI: 0.23–0.43, *p* < 0.001), West Panama 0.76 (95% CI: 0.66–0.88, *p* < 0.001), Los Santos 0.74 (95% CI: 0.57–0.95, *p* = 0.035), and Veraguas 0.84 (95% CI: 0.60–1.16, *p* < 0.001). While living in the regions of East Panama and Herrera, the risk of developing dengue hemorrhagic fever was 1.35 (95% CI: 1.08–1.68, *p* = 0.007) and 1.65 times higher (95% CI: 1.36–1.99, *p* < 0.0001), respectively. At the multivariable level, the estimates of the risk of developing dengue hemorrhagic fever remained higher for East Panama and Herrera (Table 2).

All four DENV serotypes have circulated in Panama since 1993. DENV-1 has been the most prevalent serotype, circulating during three different periods (1994–2000; 2003–2006, and 2009–2017) (Figure 1 and Supplementary Figure S2) [31]. DENV-2 circulated over 17 years during three periods (1993–1994; 1999–2005, and 2011–2017). DENV-3 was detected four times (1995; 1999–2000; 2006–2012; 2016–2017), whereas DENV-4 circulated once from 1998 to 2000. A single imported case of DENV-4 from the Dominican Republic was reported in 2015, however no autochthonous cases were detected afterwards. We observed oscillating co-circulation of multiple serotypes every 4–6 years, followed by a two-year long circulation of a dominant single serotype (DENV-1: 1996–1997, DENV-2: 2001–2002, DENV-3: 2007–2008). The re-introduction of dengue in 1993–1995 was associated with a small outbreak with DENV-2 cases reported in 1993, DENV-1 in 1994, and, finally, DENV-3 cases in 1995 (Figure 1). Detection of DENV-1 cases continued annually until 2005, with a paucity of transmission in 2001–2003. Detection of DENV-1 at the end of 2003 was associated with an increase of dengue cases in 2004–2005, as well as in 2009 after a paucity of two years. Similar trends (intense circulation followed by multiyear paucity of detection) were observed with DENV-2 and DENV-3 (Figure 1 and Table 1). 

### 3.2. Phylogenetic Analysis

Since the reintroduction in 1993, DENV has been endemic in Panama. In this study, all DENV Panamanian strains analyzed were from autochthonous cases, unless otherwise specified. 

#### 3.2.1. DENV-1 Phylogenetic Analysis of Panamanian Isolates

**Figure 2:**
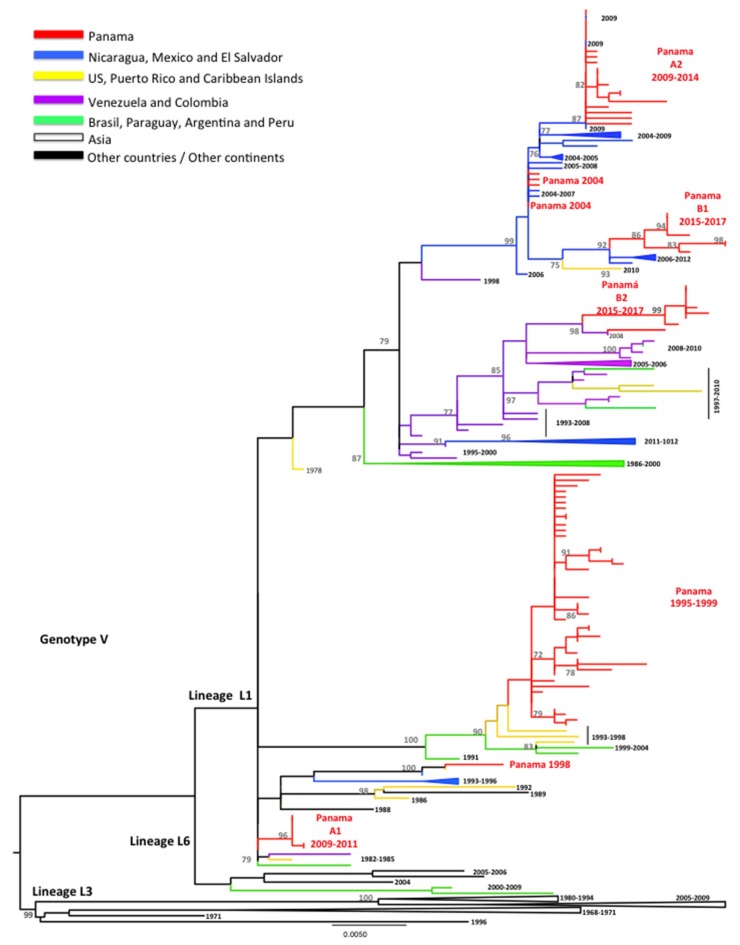
**Maximum Likelihood tree (ML) of DENV-1 envelope (E) protein genes.** The ML tree was constructed using the model General Time Reversible (GTR + I + gamma), with bootstrap values >70 (1000 replicates). Red color represents DENV-1 Panamanian strains (*n* = 93).

DENV-1 has been the main circulating serotype in Panama (Figure 1 and Supplementary Figure S2). DENV-1 serotype has five genotypes (I to V) [15,32]. DENV-1 genotype V has been divided into three lineages (L1, L3, L6), with L1 and L6 being the most described in the Americas [16,32,33,34,35]. All sequenced Panamanian DENV-1 isolates were from genotype V, and most of them had signature amino acids of L1 [34,35], although they could be grouped into different clusters (Figure 2).

During the introduction of DENV-1 in 1994, Panamanian DENV-1 strains from 1995–1999 were grouped into one cluster closely related to isolates detected previously (1993) in Puerto Rico (Figure 2). Only one Panamanian isolate from 1998 clustered with Caribbean and Central American strains (Costa Rica 1993 and Puerto Rico 1996), suggesting possible co-circulation of two clusters of DENV-1 from L1, genotype V. However, several Panamanian DENV-1 strains sampled in 2004 were related to Nicaraguan and Mexican strains sampled in 2004–2006 and 2006–2008, respectively, and contained an amino acid substitution (M297T), which was previously described in L6 [16,32,33,34,35]. 

Following the 2009 DENV-1 outbreak, Panamanian strains were grouped into two separate clusters (PA A1 2009–2011, PA A2 2009–2014), which were replaced in 2015 by strains belonging to clusters PA B1 2015–2017 and PA B2 2015–2017. The Panamanian strains of cluster PA A1 2009–2011 were related to strains sampled in South America in 1982–1986 and the Caribbean in 1991–2004. Cluster PA A2 2009–2014 contained Central American strains (Nicaraguan 2009) characterized by the substitution M297T, as described for the PA 2004 sequences. The PA B1 2015–2017 cluster was also characterized by this M297T substitution and was related to strains sampled in Central America (Nicaragua 2006–2012) and Mexico. All PA B2 2015–2017 were related to strains isolated in Colombia and Venezuela in 2004–2008 [16,32,33,34,35]. 

#### 3.2.2. DENV-2 Phylogenetic Analysis of Panamanian Isolates

**Figure 3:**
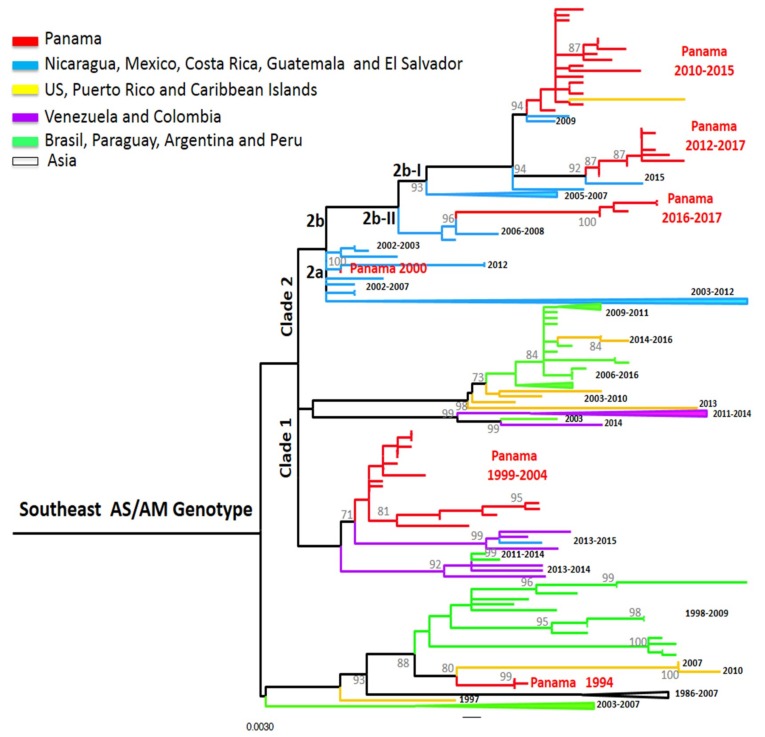
Maximum-likelihood (ML) consensus tree of the Envelope (E) protein gene of DENV-2. The ML tree was constructed using the model General Time Reversible (GTR + I+gamma), showing bootstrap values >70 (1000 replicates). Blue color represents DENV-2 Panamanian strains (*n* = 53).

DENV-2 has six genotypes corresponding to geographical regions of detection: Sylvatic, American, Cosmopolitan, Asia I, Asia II, and Southeast Asian/American (AS/AM) genotypes [15]. The gradual and complete replacement of the American genotype by the AS/AM genotype since the 1980s has been associated with more severe outbreaks [19,22,36]. DENV-2 circulation was reported on three occasions in Panama. All sequences of Panamanian DENV-2 isolates were from the AS/AM genotype (Figure 3), even during the first introduction of DENV-2 in the country in 1993–1994, although American genotype strains were still detected in the neighboring country of Costa Rica in 1994 [37]. Panamanian DENV-2 strains from 1994 clustered together alone, close to strains from the Caribbean (Puerto Rico), inside a bigger cluster containing South American strains (Peru and Brazil) (Figure 3). DENV-2 in 1994 was quickly replaced by DENV-1 and DENV-3 (Figure 1 and Supplementary Figure S2). 

DENV-2 was detected again in Panama from 1999 to 2005 and was the only detected serotype from 2001–2002. DENV-2 isolates from 1999–2004 grouped in a cluster divided into two smaller clusters, related to Colombian strains within the AS/AM clade 1 [18] (Figure 3). However, the strain PA/DENV-2/GMI482608/2000 was closer to strains sampled in Mexico, Guatemala, and El Salvador. This suggests that during that period, DENV-2 from two different clusters were co-circulating within Panama, suggesting separate introductions from these regions.

DENV-2 was not detected again in Panama until 2010 (Figure 1). During the Pan-American 2010 outbreak, neighboring countries Colombia and Costa Rica reported DENV-2 and DENV-1-3 in circulation, respectively [36]. During that year, Panama did not show an increase in cases, but reported the detection of DENV-2, with co-circulation of DENV-1 and DENV-3, like Costa Rica. In Panama, this re-emergence of DENV-2 was associated with an important epidemic event one year later [21]. DENV-2 isolates PA 2010-2015 clustered together with a Puerto Rican strain from 2013 and belonged to the AS/AM genotype sub-clade 2b-I (following [18] classification of Central American strains). They were genetically close to viruses described in Central America and Mexico from 2006–2011, which were also associated with outbreaks [18]; however the PA 2010–2015 and the PR 2013 had a substitution (A50T) in the E protein that might be associated with antibody escape [21]. Since 2012, some strains could be regrouped within another cluster, PA 2012–2017. These strains were closer to strains from Central America (El Salvador 2015) and were also in the sub-clade 2b-I, but did not harbor the substitution A50T [18]. Strains from clusters PA 2010–2015 and PA 2012–2017 co-circulated for three years, followed by strains that were grouped in a third cluster, PA 2016–2017. These were related to Nicaraguan strains from 2006–2008 from sub-clade 2b-II carrying the non-silent mutation leading to the substitution of M492V in the E protein, a signature of this sub-clade [18]. These results suggest a higher degree of diversity of DENV-2 these last years with overlap and replacement between clusters of the same sub-clade (PA 2010–2015 and PA 2012–2017) and then co-circulation of two sub-clades at the same time (2b-I and 2b-II). 

#### 3.2.3. DENV-3 Phylogenetic Analysis of Panamanian Isolates

**Figure 4:**
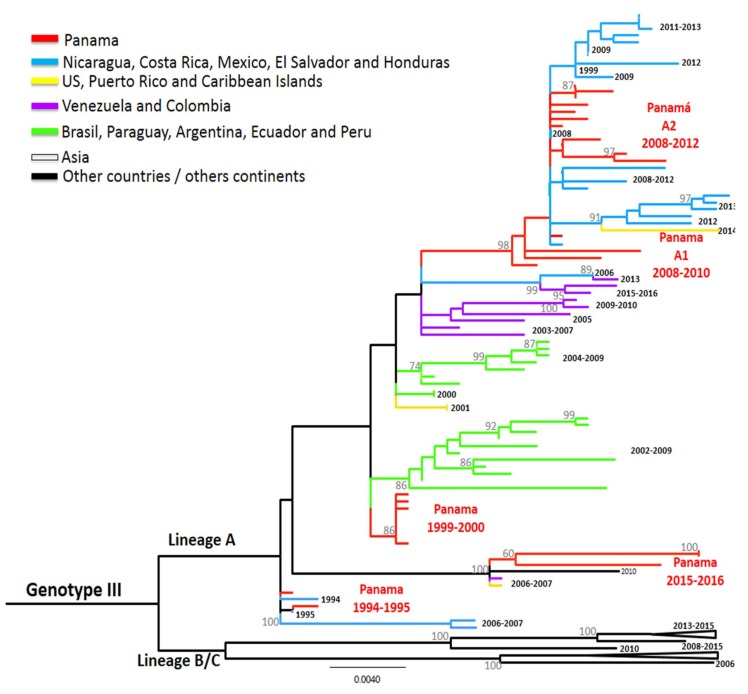
Maximum-likelihood (ML) consensus tree of the Envelope (E) protein gene of DENV-3. The ML tree was constructed using the model General Time Reversible (GTR + I + gamma), showing bootstrap values >70 (1000 replicates). Green color represents DENV-3 Panamanian strains (*n* = 26).

The genetic diversity of DENV-3 strains allowed for the division into five distinct genotypes (I-V) [15]. The appearance and dissemination of DENV-3 genotype III from Asia to the Americas have been linked to severe disease epidemics [38]. DENV-3 circulation has been reported four times in Panama, each time from genotype III strains (Figure 4), confirming previous studies stating that the first reintroduction of DENV-3 in Panama was from that genotype [13]. We looked at the amino acids previously described as having higher-scoring amino acid substitutions in DENV-3 E protein, such as residue 329 (domain III, at the surface of the protein) and between residues 380 to 385 [39], described in DENV-3 strains circulating in neighbor countries [40]. In position 329, Panamanian DENV-3 sequences, independently of the year of isolation, have a Valine residue, similar to sequences from Colombia, Venezuela, Peru, and Ecuador, so they all belonged to genotype III cluster A, following Ramirez et al.’s classification [39,40,41] (Figure 4). These first strains detected in Panama in 1994–1995 were imported cases and were similar to sequences circulating in Central America (Nicaragua, Honduras) during these years. 

The PA strains that caused the few dengue cases from 1999–2000 clustered together and were related to strains circulating at the same time in South America (Ecuador 2000), but also to others detected some years later in Mexico and South America (Figure 4). 

DENV-3 was detected again between 2006 and 2012 (Figure 1), and strains from these years were grouped into two clusters (Figure 4). PA A1 2008-2010 was closely related to strains that circulated in Nicaragua during the same years and to strains detected later in Central America and the Caribbean islands. Similarly, the second cluster PA A2 2008–2012 was also related to strains sampled in Central America during this timeframe. DENV-3 was not detected in 2013–2014, however it caused a few cases between 2015 to 2017. Notably, strains isolated and sequenced from these cases were grouped in the same cluster PA 2015–2016, which were closely related to strains from China, Puerto Rico, and Venezuela.

#### 3.2.4. DENV-4 Phylogenetic Analysis of Panamanian Isolates

**Figure 5:**
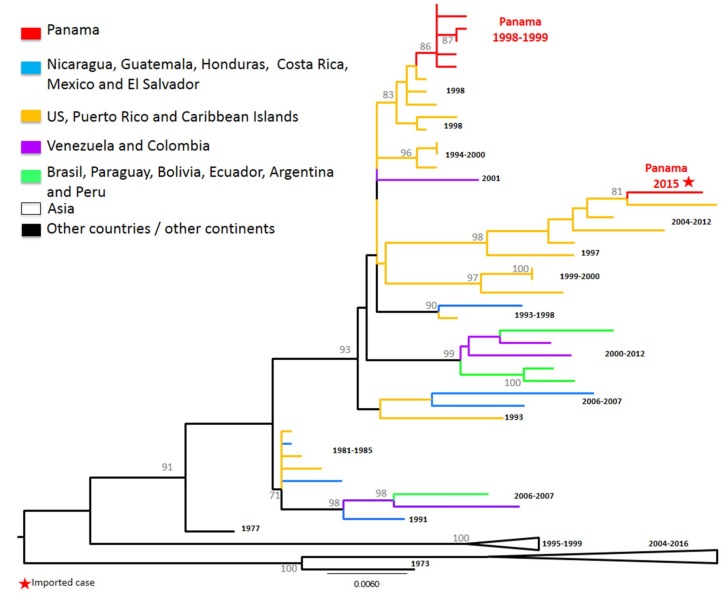
**Denv-4 phylogenetic tree.** Maximum Likelihood tree (ML) of Dengue virus 4 complete envelope (E) protein genes. The ML tree was constructed based on the model Hasegawa–Kishino–Yano, with bootstrap values (1000 replicates). Purple color represents DENV-4 Panamanian strains (*n* = 7).

DENV-4 contains three genotypes (I, II, III), representing strains sampled from the human transmission cycle, as well as the ancestral Sylvatic genotype (representing extant strains sampled from the sylvatic transmission cycle) [15]. DENV-4 has circulated autochthonously in Panama once between 1998 and 2000, although it has been circulating continuously in neighboring countries like Colombia since its reintroduction in 2000 [8]. DENV-4 detected in Panama was Genotype II and these sequences clustered with Caribbean strains (Figure 5). 

The second time DENV-4 was detected in Panama, it was an imported case in 2015 from the Dominican Republic and, as expected, it grouped with strains previously circulating in the Caribbean islands from 2010–2014. DENV-4 was not detected in other dengue cases that year nor the years after. The translated E protein obtained for each sequence showed that both clusters containing strains detected in Panama had the changes observed in Genotype II group I in Puerto Rico from 1985–1987 [42]: M163T and I351V. 

## 4. Discussion

This study analyzes the epidemiology and phylogenetic characteristics of 25 years of dengue circulation in Panama. After DENV established its endemicity in 1993, the number of suspected, confirmed, and severe/fatal dengue cases has increased steadily, mainly since 2009. Interestingly, since 2009, at least two serotypes and frequently, three serotypes have been circulating every year, suggesting that the continuous co-circulation of different serotypes might increase the probability of secondary infections and dengue severity [22,43]. This co-circulation of serotypes is similar to what has been described throughout the Americas [8,10,44]. However, Panama accounted for only 1.9% of cases in the region including the Central America isthmus and Mexico [9]. Despite several outbreaks (1995, 2005–2006, 2009, 2011, 2013–2014), dengue cases in the country always remained below the average of the region. Even neighboring countries, Costa Rica and Colombia, registered 5.5 and 18.5 times more cases than Panama during our study period, despite the constant interchange of migrants and merchandise, similar cultural characteristics, and similar demographics. Future studies are needed to determine if this difference in case numbers is due to less dengue infection associated with a strong vector control program, fewer persons attending health institutions, or underreporting of suspected dengue cases. 

### 4.1. Epidemiological Characteristics 

Our results demonstrate that Panama has a strong level of laboratory support, with the highest percent of dengue laboratory confirmation in the region. Since 1999, Panama has maintained a 50% laboratory detection and confirmation rate of dengue cases. We have also reinforced test detection during outbreaks, as in 2011 when laboratory detection reached 80.6% of reported cases. For comparison, WHO recorded for Central America and Mexico that 18.4%, 10.8%, 9.1%, and 13% of cases were laboratory confirmed between 2014 and 2017 [9], while Panama, during the same time period, recorded 57%, 58.9%, 65.9%, and 64.3% laboratory confirmed cases. 

During these 25 years of dengue in Panama, incidence rates have typically been higher among adults; we observed high incidences in the 30–59 years old group, mainly until 2009. However, more recently, there has been a trend toward infections in younger age groups with higher incidences for the 10–19 years-old age group. The same pattern has been observed in some Brazilian cities [45] [46,47], where the incidence rates have increased in younger populations. A different trend was observed in Colombia, where high incidence was reported in individuals 5–14 years of age in 2003, however transmission was highest since 2011 in the >45 years-old age group [48]. 

We observed no significant difference in risk for developing hemorrhagic dengue between females and males. The distribution of dengue by gender appears to be as variable between regions or cohorts. In French Guiana, men appeared to be slightly more affected than women in a ratio 0.99–1.22; in contrast, a hospital-based study showed that three years later, women were more affected than men in a ratio of 0.90 to 0.72 [49]. Others reported that females of all ages were at higher risk of developing severe symptoms in South America [50]. The reasons for these differences are not completely understood, however viral and host genetics, as well as local epidemiological characteristics, seem to influence which gender and age groups are more susceptible to disease and severity during an outbreak [44,51].

Hospitalization was not significantly associated with age in Panama, although more than 19% of hospitalized cases were from the 11–20 years old reference age group. However, there is a higher risk (1.36 times) of developing dengue hemorrhagic fever in the 0–10 age group compared to the reference group, similar to what has been observed in southeast Asia [12,44,50,52]. Our multivariate analysis showed that the elderly (>71 years old) also had a high risk (1.33 times) of developing hemorrhagic dengue, similar to other countries in the region [8]. This could be due to the higher level of chronic diseases, co-morbidities, as well as poorer immune responses in the elderly group [53,54]. Living in the Herrera province or the East Panama region conferred a higher risk of developing hemorrhagic dengue compared to the reference region, the Panama Metropolitan area, the region with the largest population and with a higher number of dengue cases. This could be explained by the fact that Herrera has an aging population [55] and the East Panama region has had recent urbanization and illegal settlements in what used to be swamps, mangroves, and forest areas without regular water services and garbage disposal. Moreover, in this region, health institutions were only recently made available.

#### Dengue Outbreaks in Panama

During the 2005 dengue outbreak, hospitalized cases represented 1.4% of total cases. However, our study shows that they increased the next year to 11.4% and were maintained at an average of 8.3% in subsequent years. The cost of a hospitalized dengue case in Panama during 2005 was calculated to be $1065 per case [56], one of the highest in the Central America and Mexico region during the 2000–2007 period [57]. Given that both the number of hospitalized dengue cases and the cost of hospital care have increased, this surely represents an added burden in the cost of management, treatment, and control of dengue in Panama, which needs to be evaluated. 

The case fatality rate (CFR: dengue death/total dengue cases) has been highly variable during the 25 years of dengue surveillance in Panama (CFR: 0% to 0.49%). In 2011, the CFR associated with the reintroduction of DENV-2 in the country was 0.44%, however the CFR calculated over DHF cases was 44.7% (17 deaths/38 DHF cases x 100) [21]. A year before, during a Pan-American outbreak with a particularly high DENV-2 prevalence, the CFR (deaths/DHF) was generally between 0% and 4% in most countries, with 2.6% being the median in the Americas. Even as Colombia and Honduras registered their worst dengue epidemics at that time, their CFR was around 2.3% and 2.5%, respectively [36]. In Panama, this high CFR could be the result of several factors, which include poor participation of community members in vector control, seeking medical care late in the course of clinical illness, co-circulation of other viruses (e.g., H1N1 and PTV in 2009, CHIKV since 2014, ZIKV since 2015) [58,59,60,61,62,63,64] that may have complicated accurate differential diagnosis, thus resulting in the under-reporting of dengue cases and a decrease in the detection of severe dengue cases, and artificially increasing the CFR (death/DHF). Finally, this increase in DENV-related deaths could also be correlated with the fact that cumulated dengue cases, due to virus endemicity, could have increased the probability of secondary infections and thus the probability of severity [22,65]. Although dengue severity depends partly on host genetics and immunity, it has also been associated with virus genetics (serotypes and genotypes) [12,22,66]. The recirculation of DENV-2 in 2010–2011, after the co-circulation of DENV-1 and DENV-3, was associated with a severe outbreak in 2011 [21]. This DENV-2 cluster has previously been associated with severe outbreaks in Nicaragua [22]. However, we were not able to link severity with a specific serotype in our study because the viral sequences from the most severe or fatal cases were not available.

### 4.2. Genetic Characteristics of Panamanian Dengue Strains

In our study, the genetic characteristics of Panamanian dengue strains were analyzed from isolates and not direct from sera samples. Indeed, only isolates were available from 1993-2007, moreover the sample amount from 2008-2017 sera was not enough for direct sequencing. As bottleneck selection and specific mutations induced by more than five passage in Vero cells have been described [67], our isolates all have less than two passages to minimize the introduction of these mutations. 

As in most countries of Central and South America [19], all four DENV serotypes have circulated in Panama. However, co-circulation of all serotypes in Panama was only documented for a two year period in 1998–2000, in contrast to other countries of the Americas, where it is more common to have all four serotypes in co-circulation [8]. Patterns of dengue serotype replacement in Panama are similar to other countries in the region [9,19] and seem to follow a cyclic pattern of re-introduction every 4 to 5 years, as in Costa Rica [37] and Trinidad and Tobago [68], with a predominant serotype dominating for 2–3 years [19]. However, in Panama over the past 25 years, only one genotype per serotype appears to dominate transmission, contrasting with the co-circulation and replacement of genotypes described in Central and South America [16,18,19,22,34,35,37,48]. Co-circulation and replacement of different clades within a genotype was clearly observed in Panama and was similar to what has been described elsewhere in Central and South America. Often when a serotype has re-emerged, circulating strains belong in a new cluster and/or lineage compared to strains that circulated previously, consistent with a pattern of intense diversification (“boom and bust” period) followed by lineage extinction (“pruning” period) and clade replacement. 

In some occasions, Panamanian strains were monophyletic, grouped alone in a cluster without sequences from other countries. This was observed during the first dengue reintroduction in the 1990s for DENV-1, DENV-2, and DENV-4. The grouping of PA DENV strains in consecutive years as a monophyletic cluster was observed in a few cases, suggesting local persistence (endemicity) for 2 to 6 years: DENV-1 PA A1 2009–2011, PA B1 2015–2017, DENV-2 PA 2012–2017, PA 2016–2017, DENV-3 PA 1999–2000. It will be necessary in the future to include additional sequences, if they become available, from other countries of the Central American and Caribbean region to demonstrate that our monophyletic clusters consisting of only Panamanian strains are not due to an underrepresentation of genetic diversity in the region. 

Moreover, for all serotypes, PA strains that were sampled in the 1990s were grouped with strains sampled from countries across the Americas and the Caribbean (mainly Puerto Rico). However, DENV 1-3 PA isolates, starting in 2000, were grouped in clusters with strains sampled mainly in Nicaragua and Mexico. This could suggest a sustained endemicity with exportation to other countries in the region, or a two-way exchange of strains between hyperendemic countries of the region; this pattern was described previously in Asia [69]. Independently of these scenarios, our results suggest high DENV diversity with exchange across the Americas, even if the most common exchange has been with Caribbean islands and Central America. Furthermore, DENV-1 PA B2 2015-2017 grouped with Colombian and South American strains, suggesting a wider exchange of strains inside genotype V between regions (Caribbean, South America, or Central America) than previously described [16,19]. Our data suggest that Panama participates in the exchange of DENV in the Americas, however given the information available, for most exchanges, the direction and year of exchange cannot be determined. Due to a lack of previous DENV sequences from Panama, the country has been absent from most molecular epidemiolocal and spatiotemporal phylogenetic studies of DENV in the Americas [19,20]. Future phylodynamic and phylogeographic studies with complete genome sequences from Panama and the region are needed to gain insights into DENV movements across the region. 

The co-circulation of more than one cluster for a serotype was observed mainly after 2009, and generally strains from one cluster were detected during a longer period of time than another cluster. For example, in 2009, two DENV-1 clusters, PA A1 2009–2011 and PA A2 2009–2014, overlapped, but PA A2 had maintained circulation for three more years. In 2015, again two DENV-1 clusters co-circulated, PA B1 2015–2017 and PA B2 2015–2017, but as in 2017, it is still unknown whether they will be replaced by another. Both co-circulating clusters had the amino acid L1 signature in the E protein, however PA B2 2015–2017 had one substitution S338L described for L6. Studies in Brazil showed that L1 has better in vivo replication in mosquitoes than L6; however, L6 has reduced antigenicity, weak B and T cell stimulation, and high viremia in humans, allowing transmission to vectors and suggesting an increased epidemiology fitness of L6 versus L1, explaining its dominance in that country [35]. Future studies will be needed to obtain the complete genome sequences of the Panamanian strains to detect lineage-specific mutations in order to better understand the viral and host-specific factors that influence the transmission dynamics of DENV circulation in Panama. 

For DENV-2, after 2010, three clusters overlapped PA 2010–2015, PA 2012–2017, PA 2016–2017, with the strains from the PA 2012–2017 cluster replacing the ones from PA 2010–2015 cluster after four years of co-circulation. We cannot determine whether cluster PA 2016–2017, with the E360G substitution in the E protein (changing a negatively-charged a.a. to a neutral one), will replace the PA 2012–2017 cluster or not, as the properties of this substitution have not yet been analyzed. Co-circulation of two clusters was observed only once for DENV-3 in 2008 with PA A1 2008–2010 and PA A2 2008–2012. 

Our study has some limitations. National surveillance data could significantly underestimate the true burden of disease [70] and similarly could underestimate the diversity of strains circulating. Nevertheless, it is important to remember the inferential limitations imposed by uneven spatial sampling from any national surveillance program. There is the possibility that the diversity of circulating strains in Panama is even higher than described, especially because some regions (including the indigenous regions and the border regions with Columbia and Costa Rica) send very few samples for dengue serotype and genotype identification due to cost and the logistics of transportation of the samples to Panama City. Costa Rica experienced their largest outbreak with co-circulation of DENV-1 and DENV-2 in 2007 and of DENV-1, -2, and -3 in 2013, in which their strains were closely related to other clades found in the region [37], but not with Panamanian strains, even though the neighboring countries have a high rate of interchange of people. This caveat of our study is similar to previous studies that rely on a passive surveillance system which typically underestimates the true number of cases [44]. 

## 5. Conclusions

In conclusion, our epidemiological data shows that dengue is endemic in Panama, similar to other countries in the region, with an increased number of cases and severity. We also show a high genetic diversity of DENV circulating strains, with different clusters inside the same genotype for DENV-1, DENV-2, and DENV-3, suggesting an evolution towards hyperendemicity, which is also similar to other countries of the Americas [49]. The genetic diversity observed should be taken into account for the evaluation of vaccine efficacy which also needs to contemplate the different epidemiological situations in each country and region. This underscores the importance of continuing the effort of genotype surveillance and the need to strengthen clinical and epidemiological tracking to make it more representative of the entire territory to detect any introduction of new dengue strains or of any emergent arbovirus. Moreover, performing complete genome sequencing will be needed to identify the presence of substitutions in different viral proteins that could play a role in viral fitness, immune response, and/or epidemiological fitness to better understand dengue viral evolution.

## Figures and Tables

**Table 1:** Dengue cases, incidence, and case fatality rate in Panama from 1993 to 2017.

Year of Circulation	Total Dengue Cases	Classic Dengue	Hemorrhagic Dengue	Dengue Death Cases	Hospitalized Cases	Laboratory Confirmed Cases	Dengue Incidence ^#^	CFR ^$^
Total	67,834	65,860	1974	78	4254	39,254		
1993	14	14	0	0	*MD	*MD	0.5	0
1994	790	790	0	0	*MD	*MD	29.4	0
1995	3084	3081	3	1	*MD	*MD	112.3	0.03
1996	812	812	0	0	*MD	*MD	29	0
1997	2641	2641	0	0	*MD	*MD	92.3	0
1998	2802	2801	1	0	*MD	*MD	95.9	0
1999	2785	2784	1	0	*MD	1944	93.5	0
2000	317	314	3	0	*MD	264	10.8	0
2001	1605	1598	7	0	*MD	902	53.4	0
2002	768	763	5	2	*MD	518	25.1	0.26
2003	310	309	1	0	*MD	160	9.9	0
2004	412	408	4	2	*MD	217	13	0.49
2005	5489	5482	7	5	76	3012	170	0.09
2006	4326	4319	7	1	494	3694	131.7	0.02
2007	3729	3722	7	4	271	3175	111.7	0.11
2008	1461	1457	4	3	105	1292	43	0.21
2009	7469	7423	46	12	659	4507	216.5	0.16
2010	2002	1999	3	1	113	1625	54.7	0.05
2011	3882	3844	38	17	361	3129	104.2	0.44
2012	1329	1261	68	2	71	840	35.1	0.15
2013	4781	4443	338	8	453	3554	124.2	0.17
2014	5517	5022	495	4	577	3145	141	0.07
2015	3347	3097	250	3	357	1973	84.2	0.09
2016	3327	3073	254	8	263	2193	82.4	0.24
2017	4835	4403	432	5	454	3110	118	0.1

**^#^** Dengue incidence = Dengue cases/population × 100,000 inhab, **^$^** CFR = dengue death cases/dengue cases × 100, *MD = missing data.

**Table 2:** Risk factors for dengue hemorrhagic fever in Panama, generalized univariate and multivariate linear models.

Factors	Univariate Analysis			Multivariate Analysis		
	OR	95% CI	*p* Value *	OR	95% CI	*p* Value *
**Sex**						
Female	Ref.**	**Ref.**	**Ref.**	**Ref.**	**Ref.**	**Ref.**
Male	1.08	0.99–1.18	0.071	1.04	0.95–1.14	0.294
**Age group (years)**						
0–10	1.36	1.17–1.58	<0.001	1.33	1.14–1.55	<0.001
11–20	**Ref.**	**Ref.**	**Ref.**	**Ref.**	**Ref.**	**Ref.**
21–30	0.91	0.79–1.05	0.238	0.92	0.79–1.06	0.268
31–40	0.81	0.70–0.95	0.009	0.81	0.72–0.95	0.010
41–50	0.77	0.65–0.90	0.002	0.78	0.66–0.92	0.003
51–60	0.84	0.70–1.01	0.069	0.86	0.71–1.02	0.102
61–70	0.89	0.72–1.11	0.321	0.89	0.72–1.11	0.334
71–80	1.29	0.99–1.67	0.056	1.33	1.02–1.72	0.033
>80	1.36	0.94–1.97	0.098	1.32	0.91–1.91	0.138
**Regions**						
Bocas del Toro	0.84	0.71–1.00	0.062	0.85	0.71–1.01	0.077
Darien	0.83	0.61–1.14	0.263	0.81	0.60–1.11	0.212
Chiriqui	0.29	0.22–0.38	<0.001	0.29	0.22–0.39	<0.001
Cocle	0.32	0.23–0.43	<0.001	0.32	0.23–0.44	<0.001
Colon	1.04	0.84–1.29	0.707	1.04	0.84–1.30	0.666
Herrera	1.35	1.08–1.68	0.007	1.36	1.09–1.69	0.005
Los Santos	0.74	0.57–0.97	0.035	0.75	0.57–0.99	0.042
Panama Este	1.65	1.36–1.99	<0.0001	1.63	1.35–1.98	<0.0001
Panama Metro	**Ref.**	**Ref.**	**Ref.**	**Ref.**	**Ref.**	**Ref.**
Panama Oeste	0.76	0.66–0.88	<0.001	0.77	0.67–0.88	<0.001
San Miguelito	0.94	0.82–1.07	0.383	0.93	0.81–1.07	0.373
Veraguas	0.84	0.60–1.16	<0.0001	0.85	0.61–1.18	<0.0001

* *p* value: *p* < 0.05 is considered as statistically significant within a 95% CI (Confidence Interval). Ref.** Reference groups were selected using the categories with a higher number of observations. OR (Odds Ratio) was calculated compared to the reference group using the univariate analysis or the multivariate analysis model, respectively. ORs > 1 indicated a higher risk compared to the reference group, whereas ORs < 1 indicated a protective factor.

## Supplementary Materials

The following are available online at https://www.mdpi.com/1999-4915/11/8/764/s1. Figure S1: Political map of Panama. Figure S2: Circulating serotypes in Panama from 1993 to 2017. Table S1: Primers used for Dengue E sequencing. Table S2: GenBank Sequences from other countries used for the phylogenetic analysis. Table S3: Incidence of Dengue cases per age group from 1999 to 2017. Table S4: Hospitalization due to dengue disease according to age group.
