# Supplementary material for: Molecular Epidemiology of Dengue in Panama: 25 Years of Circulation
Source: Viruses. 2019 Aug 20;11(8):764. doi: 10.3390/v11080764 (PMC6724401; doi:10.3390/v11080764)
Supplement: Supplementary file 1 [file viruses-11-00764-s001.pdf]

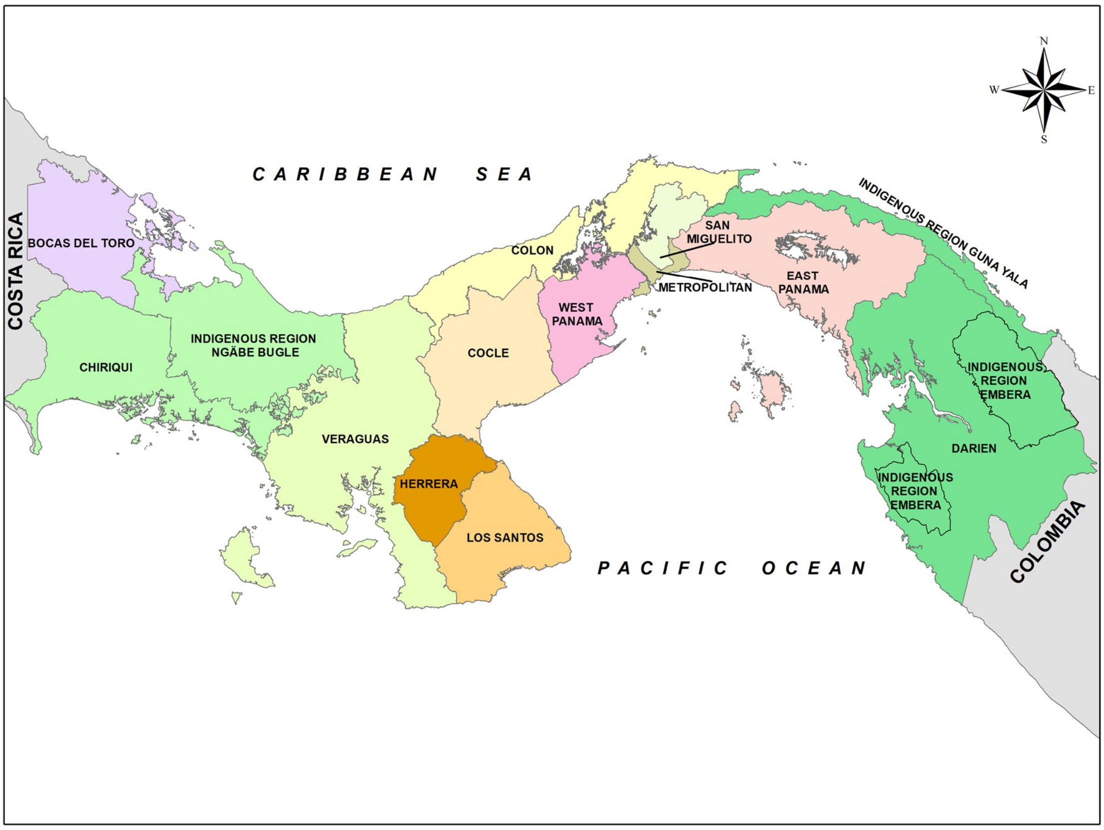

Supplementary Figure 1. Political map of Panama. Map of Panama showing the political division of the Province and Indigenous regions. The indigenous regions analyzed together with their neighbour provinces are shown in the same color (light green: Chiriqui province and Ngäbe Buglé Indigenous region; dark green: Darien provinces with the Indigenous regions Guna Yala and Emberá). Panama province is divided in four health areas by MINSA: San Miguelito, West Panama, East Panama and Metropolitan area.

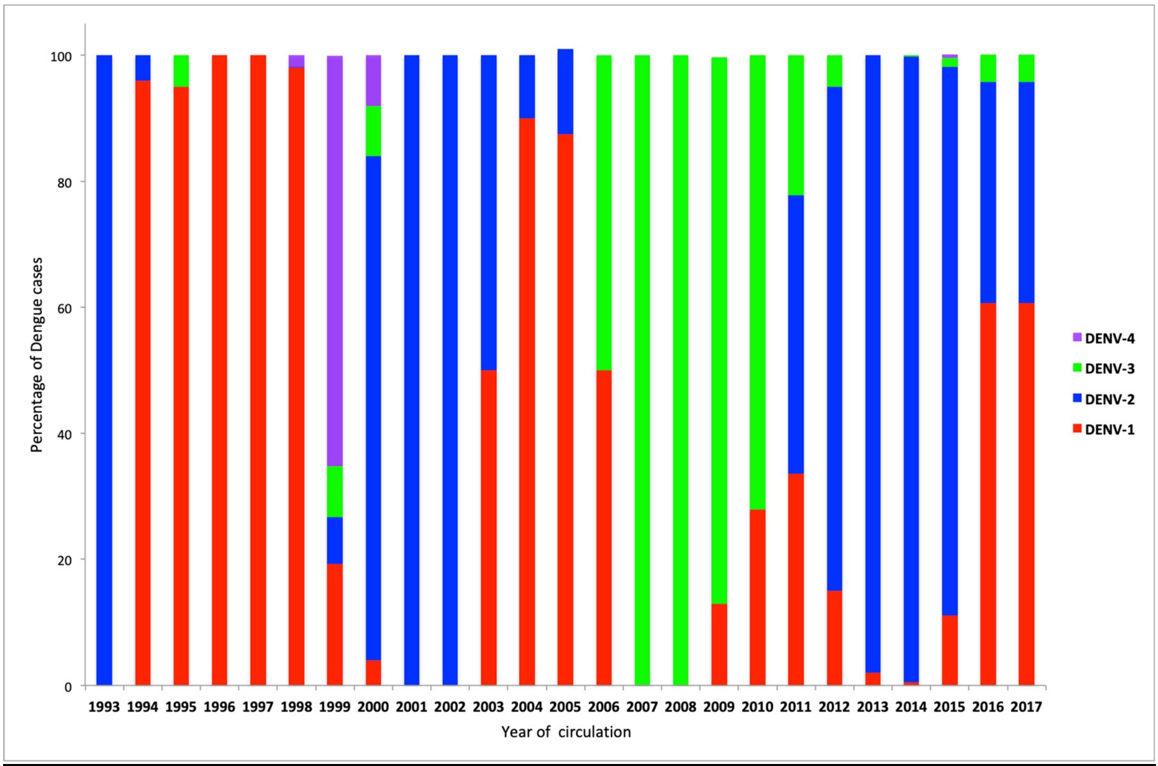

Supplementary Figure 2. Circulating serotypes in Panama from 1993 to 2017. The graph represents the percentage of circulation of serotypes from 1993 to 2009, when ICGES received all the Dengue acute samples from the country. From 2010 to 2017, ICGES received around 10% of the acute samples of each region for dengue serotype surveillance, so the percentages are representative. In the graph DENV-1 is represented in red, DENV-2 in blue, DENV-3 in green, DENV-4 in violet.

Supplementary Table 1. Groups of primers used to sequence the E gene protein of Panamanian Dengue strains. Primers to amplify the complete gene (approximate 1,800 base pair) were used from Carrillo-Valezo et al. 2010. Inner sequencing primers were specific designed for this study.

Primer name	Sequence
**D1-EIF***	AACAAGARCYGARACRTGGATGTC
**D1-E512f**	AACACCTCAAGCTCCCACGTC
**D1-E614r**	CCATTGTTTGTGGACGAGCCATG
**D1-E953f**	GTGGCTGAGACCCAGCATGG
**D1-E4R***	YARTTCATTTGATATTTGYTTCCACAT
**DENV-2-env-S3***	ACACCATAGGRACGACRYATTT
**D2-E613r**	CTGTGCACCAACCAAGC
**D2-E560f**	ACGATGGAGTGCTCTCC
**D2-E1109f**	CCAGTCAACATAGAAGCAG
**DENV-2-env-R3***	CCRCTGCCACAYTTYAGTTCT
**DEN3-E2-S***	GARAARGTAGARACATGGGC
**D3-E548f**	TGGAACCCTTGGGCTAGAATG
**D3-E610r**	CCATTGTCTGTGTACCATCCA
**D3-E1001f**	GATGCACCTTGCAAGATTCC
**DEN3-E2-R***	TCNGCYTGRAAATTTGTATTGCTC
**DEN4-env-S2***	TGGATACTYAGAAAYCCAGGART
**D4-E455f**	TGGAGACACCCATGCAGTAGG
**D4-E665r**	CCTCTGATGTATCTGCTCCTG
**D4-E956f**	GGCAGAAACACAGCATGGGAC
**DEN4-env-R2***	ACTCTGGTTGYAATTMGTACTG

*Ref: Carrillo_Valenzo E., 2010. Arch Virol 155:1401-1412. DOI 10.1007/s00705-010-0721-1

Supplementary table 2. Gene Bank sequences selected to align and construct phylogenetic trees with the Panamanian Dengue sequences. Gen Bank sequences were chosen by country, year and specific genotype for each serotype, dependent of Panamanian Dengue genotype. Country code bases on ISO 3166/2.

Gene bank sequences used for phylogenetic analysis			
**DENV-1**	**DENV-2**	**DENV-3**	**DENV-4**
NI/DENV-1/FJ024485.1/2005	PE/DENV-2/JX051780/2008	HN/DENV-3/KY851592/2013	HN/DENV-4/GU586124/2007
NI/DENV-1/FJ432720.1/2005	PR/DENV-2/KC812279/2006	HN/DENV-3/KY851593/2013	BR/DENV-4/KP638365/2012
US/DENV-1/FJ410183/1993	PE/DENV-2/JX051770/2001	HN/DENV-3/KY851603/2013	VE/DENV-7/FJ882592.1/2008
PE/DENV-1/AF425626.1/1991	PY/DENV-2/KF419415/2011	NI/DENV-3/KF973480/2012	CO/DENV-4/GQ868581.1/2001
CO/DENV-1/GQ868570.1/2008	BR/DENV-2/GQ368174/1998	NI/DENV-3/KF973481/2011	GP/DENV-4/DQ390320.1/2004
NI/DENV-1/JF937644/2009	BR/DENV-2/GQ368165/2008	NI/DENV-3/HQ166031/2009	PR/DENV-4/AY152036.1/1998
MX/DENV-1/GQ868504.1/2007	PR/DENV-2/JQ013409/2007	NI/DENV-3/HQ166032/2009	PR/DENV-4/AY152048.1/1998
MX/DENV-1/HM171562.1/2006	PR/DENV-2/KM279408/2013	NI/DENV-3/JF920406/2009	US/DENV-4/FJ882598.1/1998
NI/DENV-1/JQ287666/2009	MX/DENV-2/KM279430/2011	NI/DENV-3/KF973479/2012	HT/DENV-4/KT276273/2014
NI/DENV-1/FJ547089.1/2005	MX/DENV-2/KM279425/2011	CR/DENV-3/JF804039/1995	PR/DENV-4/JX476036/2004
MX/DENV-1/HM171558.1/2006	MX/DENV-2/KM279424/2012	NI/DENV-3/KF921929/2010	DO/DENV-4/JF804053/1997
NI/DENV-1/FJ898437.1/2004	CN/DENV-2/JN029821/1986	NI/DENV-3/KF955488/2008	PR/DENV-4/JX476032/2010
MX/DENV-1/KJ189342/2009	PE/DENV-2/JX051798/2011	NI/DENV-3/KF973476/2011	PR/DENV-4/KF809758/2012
SV/DENV-1/EU448414.1/2006	PY/DENV-2/JX051814/2010	NI/DENV-3/FJ873812/2008	DO/DENV-4/AF326573.1/1981
NI/DENV-1/EU596501.1/2004	BR/DENV-2/KJ147102/2006	SV/DENV-3/JX891664/2012	BB/DENV-4/AY152368.1/1999
MX/DENV-1/GQ868539.1/2008	BR/DENV-2/KJ147098/2006	SV/DENV-3/JX891665/2012	MS/DENV-4/AY152369.1/1994
MX/DENV-1/GQ868501.1/2007	BR/DENV-2/KJ147096/2006l	HN/DENV-3/KY851602/2013	TT/DENV-4/JF804059/2000
NI/DENV-1/FJ024483.1/2005	BR/DENV-2/KJ147097/2007	HN/DENV-3/KY851604/2013	EC/DENV-4/GQ139572/2006
MX/DENV-1/GU131957/2006	MX/DENV-2/HM171546/2003	HN/DENV-3/KY851605/2013	PE/DENV-4/GQ139558/2008
US/DENV-1/JQ675358/2010	SA/DENV-2/JX891662/2012	NI/DENV-3/HM181972/2009	CO/DENV-4/KC009639/2000
NI/DENV-1/FJ562104.1/2006	PE/DENV-2/JX051778/2007	NI/DENV-3/KF971708/2010	EC/DENV-4/GQ139575/2000
NI/DENV-1/GQ199857.1/2008	PE/DENV-2/JX051776/2002	US/DENV-3/KM458191/2014	HN/DENV-4/|AY152379.1/1991
NI/DENV-1/KF973457/2012	PE/DENV-2/JX051794/2011	CO/DENV-3/HM030549/2003	CR/DENV-4/AY934757.1/1993
MX/DENV-1/GQ868498/2006	PE/DENV-2/JX051785/2010	CO/DENV-3/KX926475/2007	MQ/DENV-4/DQ390319.1/2004
VE/DENV-1/GU056032.1/1998	BO/DENV-2/JX051807/2010	MX/DENV-3/HM171538/2006	GF/DENV-4/DQ390323.1/2005
VE/DENV-1/AF425635.1/1995	EC/DENV-2/JX051799/2000	CO/DENV-3/KY851577/2013	PR/DENV-4/AH011958.1/1998
VE/DENV-1/GU131833.1/2000	BO/DENV-2/JX051806/2007	CO/DENV-3/MH544647/2015	US/DENV-4/FJ882595.1/1998
VE/DENV-1/FJ639735.1/1997	BO/DENV-2/JX051810/2010	CO/DENV-3/MH544651/2016	BS/DENV-4/AY152364.1/1998
VE/DENV-1/FJ639735.1/1997	PY/DENV-2/JX051813/2010	CO/DENV-3/KX926477/2009	GF/DENV-4/DQ390322.1/1993
NI/DENV-1/KF973455/2012	PE/DENV-2/JX051768/2000	CO/DENV-3/KX926489/2010	MX/DENV-4/DQ341213.1/1985
VE/DENV-1/AF425637.1/1993	PE/DENV-2/JX051767/2000	CO/DENV-3/FJ189468/2005	CO/DENV-4/JF804052/2006
VE/DENV-1/AF425636.1/1994	PE/DENV-2/JX051783/2009	CO/DENV-3/HM030556/2005	MX/DENV-4/JF804055/2006
VE/DENV-1/AF425638.1/1995	PE/DENV-2/JX051781/2009	CN/DENV-3/JN029822/2010	ID/DENV-4/U18428.1/1973
VE/DENV-1/FJ850100.1/2007	BO/DENV-2/JX051803/2007	VE/DENV-3/EU932688/2007	ID/DEBV-4/U18430.1/1977

VE/DENV-1/FJ639824.1/2006	PE/DENV-2/JX051789/2009	US/DENV-3/FJ547085/2006	SG/DENV-4/AY762085.1/1995
CO/DENV-1/GQ868562.1/2005	PE/DENV-2/JX051784/2009	PE/DENV-3/KJ189255/2002	MY/DENV-4/FM986664.1/1999
BR/DENV-1/FJ850093.1/2008	BO/DENV-2/JX051801/2003	PE/DENV-3/KJ189266/2004	SG/DENV-4/KY921910/2016
CO/DENV-1/KJ189304/2005	BO/DENV-2/JX051808/2006	PE/DENV-3/KJ189292/2009	TT/DENV-4/AY152382.1/1982
CO/DENV-1/GQ868563/2006	GT/DENV-2/GU586492/2007	PE/DENV-3/KJ189294/2006	JM/DENV-4/AY152384.1/1983
VE/DENV-1/FJ850104/2008	TT/DENV-2/JF804038/1997	PE/DENV-3/KJ189296/2006	SV/DENV-4/U18426.1/1983
NI/DENV-1/JF937645/2009	BR/DENV-2/GQ368176/2008	PE/DENV-3/KJ189299/2005	ID/DENV-4/EU448461.1/2004
CO/DENV-1/GQ868559/1998	CO/DENV-2/KU878575/2014	EC/DENV-3/FJ898457/2000	
VE/DENV-1/FJ639811.1/2005	CO/DENV-2/KU878574/2014	EC/DENV-3/JF804045/2000	
VE/DENV-1/GU056030.1/1997	CO/DENV-2/KU878572/2013	BR/DENV-3/GQ330473/2009	
AR/DENV-1/KC692516/2010	CO/DENV-2/KU878571/2013	BR/DENV-3/GU131860/2006	
AR/DENV-1/KC692514/2010	CO/DENV-2/KU878569/2013	BR/DENV-3/GU131861/2007	
PR/DENV-1/KJ189361/2010	CO/DENV-2/KU878565/2013	BR/DENV-3/JX669489/2003	
PR/DENV-1/KJ189350/2012	CO/DENV-2/KU878567/2013	BR/DENV-3/JX669495/2004	
VE/DENV-1/GU131832/2000	BR/DENV-2/GQ368170/200	BR/DENV-3/JX669500/2005	
VE/DENV-1/GU056031.1/1998	MX/DENV-2/HM171556/2004	BR/DENV-3/KC425219/2002	
BR/DENV-1/HQ026760.1/1986	VN/DENV-2/GU211757/2006	BR/DENV-3/FJ898446/2001	
BR/DENV-1/AF425614.1/1997	MX/DENV-2/HM171545/2003	PG/DENV-3/EU045316/2002	
BR/DENV-1/FJ384655.1/2001	DO/DENV-2/JF804031/2003	PG/DENV-3/EU045323/2006	
AR/DENV-1/AY277658.1/2004	MX/DENV-2/FJ931535/2005	PG/DENV-3/EU045321/2003	
AR/DENV-1/AY277665.1/2004	BR/DENV-2/GQ368166/2008	MX/DENV-3/FJ898442/2007	
PY/DENV-1/AF514883.2/2000	CO/DENV-2/JF804029/2007	SG/DENV-3/MG895232/2015	
TT/DENV-1/AF425631.1/1978	JM/DENV-2/JF804034/2008	TH/DENV-3/KU509303/2013	
AO/DENV-1/AF425610.1/1988	MX/DENV-2/HM171543/2002	IN/DENV-3/JQ686075/2010	
US/DENV-1/FJ478457.1/1996	BR/DENV-2/GQ368161/2007	SG/DENV-3/KX224286/2014	
CR/DENV-1/AY153755.1/1993	BR/DENV-2/GQ330472/2009	TH/DENV-3/KT758784/2015	
MX/DENV-1/DQ341192.1/1994	BR/DENV-2/GQ368163/2007	LK/DENV-3/KU509283/2006	
MX/DENV-1/DQ341194.1/1995	BR/DENV-2/GQ368158/1998	MY/DENV-3/MG895222/2014	
GD/DEV-1/AF425618.1/1977	MX/DENV-2/JF804035/2002	SG/DENV-3/JN030166/2008	
US/DENV-1/FJ562106.1/1986	VN/DENV-2/GU211748/2007		
US/DENV-1/FJ410186.1/1992	HN/DENV-2/GU586122/2007		
PF/DENV-1/AY630408.1/1989	BR/DENV-2/GQ368169/2008		
US/DENV-1/FJ410185.1/1993	MX/DENV-2/HM171550/2005		
US/DENV-1/FJ205872/1998	BR/DENV-2/GQ368159/1998		
PY/DENV-1/AB111065.1/1999	MX/DENV-2/HM171555/2002		
AR/DENV-1/AY277663.1/2004	HN/DENV-2/GU586123/2007		
BR/DENV-1/AF425613.1/1982	BR/DENV-2/GQ368173/1998		
CO/DENV-1/AF425616.1/1985	MX/DENV-2/HM171549/2003		
TT/DENV-1/AF425639.1/1986	PY/DENV-2/KF419412/2011		
RE/DENV-1/DQ285554.1/2004	HT/DENV-2/KX702403/2016		
SG/DENV-1/EU081258.1/2005	HT/DENV-2/KY415992/2014		
IN/DENV-1/EU448413.1/2006	US/DENV-2/KX702404|/2016		
BR/DENV-1/FJ850071.1/2000	CO/DENV-2/KY905139/2015		

BR/DENV-1/HM043710.1/2009	BR/DENV-2/KT438618/2014		
MM/DENV-1/AY589692.1/1996	CO/DENV-2/KX901651/2014		
SA/DENV-1/AM746220.1/1994	CO/DENV-2/KX901650/2014		
TH/DENV-1/AY732476.1/1980	EC/DENV-2/KY474314/2014		
CN/DENV-1/HQ149733.1/2009	NI/DENV-2/EU482603.1/2007		
IN/DENV-1/JQ922548/2005	NI/DENV-2/EU482691.1/2006		
SG/DENV-1/AY762084.1/1993	NI/DENV-2/EU482689.1/2006		
IN/DENV-1/JN903579/2008	NI/DENV-2/EU482748.1/2005		
CI/DENV-1/AF425620.1/1985	NI/DENV-2/FJ205885.1/2008		
NE/DENV-1/AF425625.1/1968	NI/DENV-2/FJ639833.1/2007		
IN/DENV-1/JQ922546/1971	NI/DENV-2/FJ744709.1/2008		
	NI/DENV-2/HM631866.1/2006		
	GU/DENV-2/HQ999999.1/2009		
	CR/DENV-2/KY461758.1/2007		
	SV/DENV-2/JX891663/2012		
	PR/DENV-2/KM279409/2013		
	CO/DENV-2/KX901651/2014		
	SV/DENV-2/MH253297/2015		

Country code:

NI	Nicaragua	ID	Indonesia
US	United State of America	PG	Papua New Guinea
PE	Peru	LK	Sri Lanka
CO	Colombia	MV	Maldives
MX	Mexico	MM	Myanmar
SV	El Salvador	SG	Singapour
VE	Venezuela	BR	Brazil
PR	Puerto Rico	AR	Argentina
PY	Paraguay	TT	Trinidad and Tobago
AO	Angola	CR	Costa Rica
GD	Granada	PF	French Polynesia
RE	Reunion Island	SA	Saudi Arabia
TH	Thailand	CN	China
IN	India	CI	Ivory Coast
NE	Niger	MQ	Martinique
HT	Haiti	GF	French Guyana
JM	Jamaica	EC	Ecuador
HN	Honduras	BO	Bolivia
GT	Guatemala	DO	Dominican Republic
BS	Bahamas	MS	Monserrat

Supplementary table 3. Incidence of Dengue by age groups from 1999 to 2017.

Age group (years)	**1999**	**2000**	**2001**	**2002**	**2003**	**2004**	**2005**	**2006**	**2007**	**2008**	**2009**	**2010**	**2011**	**2012**	**2013**	**2014**	**2015**	**2016**	**2017**
**<1 y**	7.14	0.00	12.58	15.29	8.30	2.76	17.86	21.93	13.68	15.03	23.20	10.91	2.71	22.85	65.61	17.46	5.37	6.72	6.73
**1-9 ys**	25.88	2.37	26.04	9.72	4.55	3.54	65.23	49.83	47.91	14.32	84.86	25.61	57.22	20.86	61.90	119.11	80.81	53.49	79.24
**10-19 ys**	73.18	6.50	41.70	20.67	7.81	10.64	176.81	125.49	90.95	38.45	227.50	55.14	112.69	41.68	123.13	144.60	94.57	108.30	151.34
**20-29 ys**	110.43	11.47	54.39	31.72	11.12	14.16	176.40	134.80	111.63	47.64	234.20	10.74	113.43	37.18	140.24	152.44	97.17	90.80	130.28
**30-39 ys**	135.20	15.89	78.90	33.57	15.78	23.04	187.70	144.62	130.88	47.74	235.26	8.86	106.94	39.24	136.54	137.86	89.70	88.29	128.86
**40-49 ys**	162.32	20.54	78.01	37.62	15.21	16.28	218.84	162.56	127.72	53.38	251.90	7.73	128.18	40.05	153.83	144.89	76.06	91.19	129.36
**50-59 ys**	102.75	17.08	64.08	30.45	5.10	16.38	234.11	192.60	159.63	58.46	265.49	9.03	133.42	35.67	162.87	160.61	79.12	81.63	114.15
**60-69 ys**	98.30	15.57	40.80	15.20	8.67	9.01	196.23	167.21	153.87	57.51	264.61	5.20	129.28	40.07	161.29	169.08	80.01	76.48	102.02
**>70 ys**	74.75	5.47	53.49	10.12	3.25	8.58	167.97	160.63	171.55	115.54	212.84	32.40	88.00	26.46	85.35	142.59	66.19	56.85	95.40

Supplementary Table 4. Percentage of hospitalized cases due to dengue infection in Panamá from 2005 to 2017.

Age group (years)	Percentage of hospitalizated dengue cases from 2005 to 2017
Total	100.0
<1 y	1.5
1-9 ys	13.0
10-19 ys	19.1
20-29 ys	15.8
30-39 ys	13.1
40-49 ys	11.2
50-59 ys	8.6
60-69 ys	7.6
>70 ys	10.0
